# Integrated Transcriptomic and Metabolomic Analyses Reveal the Mechanism by Which 5-Methoxyindole Enhances Sesquiterpenoids Production in *Atractylodes chinensis* Hairy Roots

**DOI:** 10.3390/plants15132027

**Published:** 2026-06-30

**Authors:** Beibei Shi, Xiaolu Huang, Xiao Yu, Ying Li, Renjun Mao, Zhenqing Bai

**Affiliations:** 1 Shaanxi Key Laboratory of Research and Utilization of Resource Plants on the Loess Plateau, College of Life Sciences, Yan’an University, Yan’an 716000, China; shibeibei@yau.edu.cn (B.S.); 15347789237@163.com (X.H.); 18700100760@163.com (X.Y.); 19829888976@163.com (Y.L.); mrjnwsuaf@126.com (R.M.); 2 College of Grassland Science, Inner Mongolia Agricultural University, Hohhot 010018, China

**Keywords:** *β*-eudesmol, 5-methoxyindole, terpene synthase, hairy roots

## Abstract

*Atractylodes chinensis* (DC.) Koidz is a pharmacologically significant medicinal plant that produces various bioactive metabolites, including β-eudesmol, which largely determines its medicinal quality and clinical efficacy. Treatment of *A. chinensis* hairy roots with 5-methoxyindole (5-MI), a chemical homolog of melatonin, revealed that 0.5 mmol·L^−1^ 5-MI significantly enhanced β-eudesmol production. To investigate the underlying mechanism, we performed integrated transcriptomic and metabolomic analyses. Comprehensive targeted metabolomics identified ten upregulated terpenoids among the differentially expressed metabolites following 5-MI treatment in *A. chinensis* hairy roots, including β-eudesmol. The 5-MI treatment significantly influenced the expression levels of genes associated with metabolic regulation and secondary metabolite synthesis in *A. chinensis* hairy roots, particularly promoting the upregulation of genes involved in terpenoid biosynthesis. Notably, a putative sesquiterpene synthase gene, *AcTPS1*, was significantly upregulated after 5-MI treatment and was a strong candidate gene correlated with *β*-eudesmol. *AcTPS1* belongs to the TPS-a subfamily and exhibits tissue-specific expression, with high transcript levels in root tissues, especially in one-year-old roots of *A. chinensis*. This study demonstrates that 5-MI serves as an effective elicitor, providing valuable insights for the production of medicinally active compounds and enhancing our understanding of the molecular mechanism by which 5-MI regulates plant secondary metabolism.

## 1. Introduction

Chemical elicitors are widely used to enhance the biosynthesis and accumulation of plant secondary metabolites [1]. Melatonin (MT, *N*-acetyl-5-methoxytryptamine) is a ubiquitous bioactive small molecule in plants and has been verified as a highly efficient elicitor that significantly promotes the biosynthesis and accumulation of secondary metabolites in various plant species [2,3,4]. For instance, exogenous melatonin treatment markedly increased phenolic, flavonoid, and anthocyanin contents in tomato seedlings [5] and significantly elevated the total glucosinolate content in broccoli [6]. However, the low endogenous content of melatonin in plants and its complex biosynthetic pathway limit its large-scale application as an elicitor in agriculture [7]. Therefore, screening and identifying highly efficient functional substitutes for melatonin are of great value for agricultural production and the regulation of secondary metabolism in medicinal plants. Previous studies have confirmed that 5-methoxytryptamine and 5-methoxyindole (5-MI) can be recognized by the same receptor protein in plants as melatonin. Moreover, 5-MI is characterized by a low synthesis cost and high preparation efficiency, exhibiting great potential for development and application [8]. To date, reports on the function of 5-MI have primarily focused on its antibacterial activity [7], while its role and mechanism as an elicitor of plant secondary metabolism remain unexplored.

5-Methoxyindole is a synthetic indole-based heterocyclic aromatic derivative with unique physiological activities. Industrially, it is synthesized from raw materials such as *p*-methoxyaniline, *p*-methoxynitrobenzene, 2-methyl-4-methoxynitrobenzene, naphthol, and naphtho [9,10]. As an important pharmaceutical intermediate, 5-MI is used in the synthesis of amino acids, flavors, dyes, and other substances, with a wide range of application scenarios [11]. Existing studies have revealed that the combined application of 5-MI and nano-biochar significantly enhances the plant growth-promoting effect of nano-biochar on belowground parts, resulting in a higher belowground biomass than nano-biochar treatment alone [12].

Secondary metabolites are the material basis for the clinical efficacy of medicinal plants and are core indicators for evaluating the quality of traditional Chinese medicinal materials [13]. Roots are commonly used medicinal tissues and the primary site of secondary metabolite production [14]. As the main organ for the synthesis and accumulation of secondary metabolites in medicinal plants, hairy roots are adventitious root culture systems induced by *Agrobacterium rhizogenes*, which possess the advantages of rapid growth, high genetic stability, and a strong secondary metabolite synthesis capacity, making them an ideal system for producing bioactive components of medicinal plants [15].

At present, adding chemical elicitors to hairy root cultures has become a mainstream strategy to enhance secondary metabolite yields. In our previous research, hairy roots of *Atractylodes chinensis* (DC.) Koidz were treated with melatonin and its two chemical homologs (5-MI and 5-methoxytryptamine) at different concentrations (0, 0.01, 0.05, 0.1, 0.2, and 0.5 mmol·L^−1^), and the effects on secondary metabolite contents were evaluated. The results showed that only treatment with 0.5 mmol·L^−1^ 5-MI for 7 d significantly increased the β-eudesmol content to 17.37 mg·g^−1^ DW in *A. chinensis* hairy roots, confirming that 5-MI treatment promotes the biosynthesis and accumulation of sesquiterpenoids in *A. chinensis* hairy roots [16]. However, the molecular mechanism by which 5-MI regulates secondary metabolite biosynthesis and accumulation in *A. chinensis* remains unclear.

*A. chinensis* is a perennial herb of the genus *Atractylodes* in the Asteraceae family. The rhizome of *A. chinensis* is a widely used traditional Chinese medicinal material, also named “Cangzhu”; it is known for its core effects of drying dampness, invigorating the spleen, dispelling wind, and dissipating cold, and it is commonly used to treat rheumatic arthralgia and other diseases, attracting extensive attention from the academic community worldwide [17,18,19]. While the *Flora of China* treats *A. chinensis* as a synonym of *A. lancea*, the *Chinese Pharmacopoeia* and recent phylogenomic studies support *A. chinensis* as a distinct chemotype [20,21]. Our study uses ‘Northern Cangzhu’ collected from northern China, Inner Mongolia Province, corresponding to *A. chinensis* in the traditional Chinese medicinal context. Modern pharmacological studies have verified that *β*-eudesmol is the most abundant characteristic bioactive sesquiterpenoid in *A. chinensis* and is the core component underlying its anti-inflammatory, antibacterial, and gastrointestinal function regulatory activities [22,23,24]. In recent years, market demand for *Atractylodes* medicinal materials has increased, while wild resource reserves have sharply declined. Improving medicinal material quality has thus become an urgent issue for the development of modern traditional Chinese medicine. Therefore, this study used *A. chinensis* hairy roots as research material to explore the signaling mechanism by which 5-MI induces terpenoid biosynthesis. Metabolomics was used to analyze the regulatory effects of 5-MI treatment on terpenoid accumulation, and differentially expressed metabolites (DEMs) related to terpenoid synthesis were screened via principal component analysis (PCA), orthogonal partial least squares discriminant analysis (OPLS-DA), metabolic heatmap analysis, functional annotation and classification, and metabolic pathway enrichment analysis. Transcriptomics was employed to dissect the transcriptional regulatory mechanism of 5-MI in terpenoid biosynthesis, and key enzyme genes associated with terpenoid biosynthesis after 5-MI treatment were screened through bioinformatics analysis and qRT-PCR. We now explicitly state our hypothesis: 5-MI acts as an elicitor to transcriptionally activate the MVA pathway and specific terpene synthases, leading to increased *β*-eudesmol accumulation. We then follow with two specific objectives: (1) to identify global changes in metabolites and transcripts in response to 5-MI using multi-omics and (2) to clone and characterize the key terpene synthase *AcTPS1*.

## 2. Results

### 2.1. 5-MI Treatment Significantly Increased the Content of β-Eudesmol

Based on the results of our previous study, *A. chinensis* hairy roots were treated with MT and its two chemical homologs, 5-MI and 5-MT, at concentrations of 0, 0.01, 0.05, 0.1, 0.2, and 0.5 mmol·L^−1^ [16]. The results showed that only treatment with 0.5 mmol·L^−1^ 5-MI for 7 d significantly increased the content of *β*-eudesmol to 17.37 mg·g^−1^ DW. In the present study, after treating *A. chinensis* hairy roots with the same concentration of 5-MI for 7 days, the content of *β*-eudesmol in *A. chinensis* hairy roots significantly increased to 16.91 mg·g^−1^ DW (Table 1).

### 2.2. Identification of DEMs Under 5-MI Treatment

**Figure 1:**
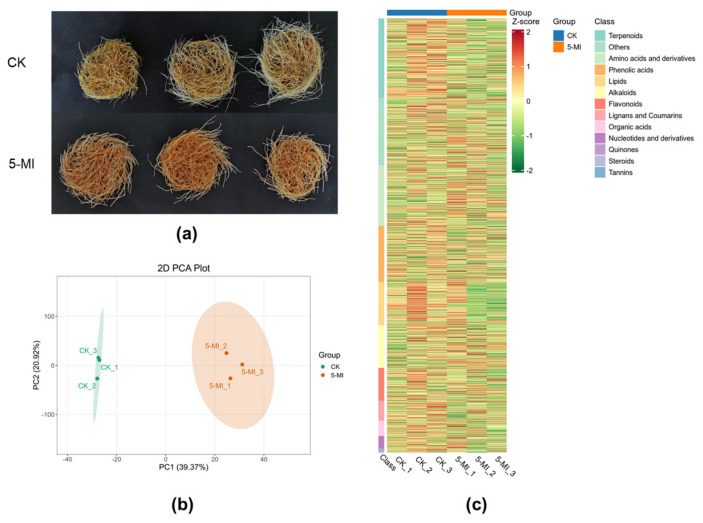
Effects of 5-MI treatment on the metabolome of *A. chinensis* hairy roots. (**a**) Schematic representation of sample collection at 7 days post-treatment (dpt) with 0.5 mmol·L^−1^ 5-MI. (**b**) OPLS-DA score plot of metabolites in the control (CK) and 5-MI treatment groups. (**c**) Classification of the 2308 detected metabolites into compound categories.

To evaluate the effect of 0.5 mmol·L^−1^ 5-MI on the metabolites of *A. chinensis* hairy roots, samples were collected at 7 dpt (Figure 1a), and a widely targeted metabolomic analysis was performed to investigate the metabolite differences between the control (CK) and the 5-MI treatment groups. At 7 dpt, a total of 2308 metabolites were detected in the CK and 5-MI treatment groups. OPLS-DA was conducted to explore the relationship between the samples and their metabolomic profiles. The results showed that all samples were within the 95% confidence interval, with a dense distribution within each group and clear separation between groups (Supplementary Figure S1). The CK and 5-MI treatment groups were clustered into two distinct groups, indicating the reliability of the metabolomic data (Figure 1b). The detected compounds were classified into flavonoids, phenolic acids, nucleotides and their derivatives, lignans and coumarins, amino acids and their derivatives, tannins, terpenoids, organic acids, lipids, alkaloids, quinones, steroids, and other categories. Among them, terpenoids were the most abundant category, with 423 metabolites detected (Figure 1c).

**Figure 2:**
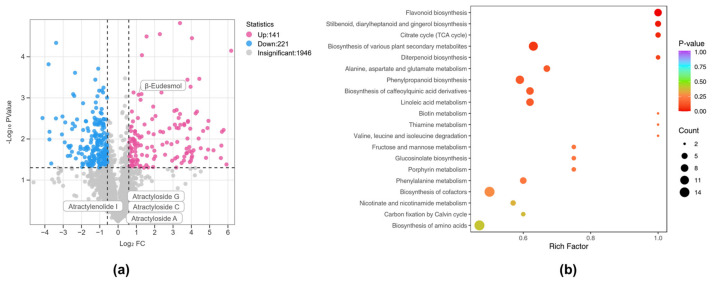
Volcano plot and KEGG enrichment analysis of the effect of 5-MI treatment on the metabolome of *A. chinensis* hairy roots. (**a**) Volcano plot of DEMs in the CK vs. 5-MI comparison. Red indicates upregulated metabolites (141), blue indicates downregulated metabolites (221), and gray indicates metabolites with no significant differences (1941). (**b**) KEGG enrichment analysis of DEMs in the CK vs. 5-MI comparison.

Based on OPLS-DA multivariate statistical analysis, the DEMs in hairy roots after 5-MI treatment were screened using criteria of VIP ≥ 1 and FC ≥ 1.5 or FC ≤ 0.75. Compared with CK, a total of 362 significant DEMs were detected in the 5-MI treatment group, including 141 upregulated DEMs and 221 downregulated DEMs (Figure 2a). Among the 141 upregulated DEMs, 10 were terpenoids, including the sesquiterpenoids dictyosporin C, alpha-curcumene, and *β*-eudesmol (Table 2 and Table S1, Supplementary Figure S2). KEGG enrichment analysis was performed on the significant DEMs (Figure 2b). The results showed that the DEMs were mainly enriched in pathways such as flavonoid biosynthesis; stilbenoid, diarylheptanoid, and gingerol biosynthesis; citrate cycle (TCA cycle); biosynthesis of various plant secondary metabolites; and diterpenoid biosynthesis. Among these, a total of three DEMs were enriched in the diterpenoid biosynthesis metabolic pathway, which is related to terpenoid biosynthesis. Therefore, 5-MI significantly affects the metabolic profile of *A. chinensis* hairy roots and induces extensive changes in metabolites.

### 2.3. Identification of DEGs Under 5-MI Treatment

**Figure 3:**
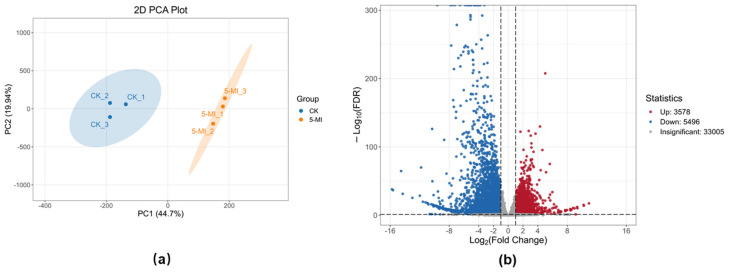
PCA and volcano plot analysis of the effect of 5-MI treatment on the transcriptome of *A. chinensis* hairy roots. (**a**) PCA score plot of transcriptomic profiles. (**b**) Volcano plot showing significant DEGs in the CK vs. 5-MI comparison.

To evaluate the effect of 5-MI on the gene transcription level in *A. chinensis* hairy roots, RNA-Seq analysis was performed on *A. chinensis* hairy roots at 7 dpt to quantify the changes in gene expression. A total of 80,437 transcripts were obtained. The Unigene sequences were aligned with the KEGG, NR, Swiss-Prot, GO, COG/KOG, and TrEMBL databases using BLAST (v2.14.0) software. After predicting the amino acid sequences of the Unigenes, HMMER (v3.3.2) software was used to align the sequences with the Pfam database to obtain the annotation information of the Unigenes. The data quality met the requirements for subsequent transcriptomic analysis (Supplementary Table S2). The PCA results showed clear separation between the CK and 5-MI groups, indicating high reliability of the transcriptomic data (Figure 3a). Compared with CK, a total of 3578 genes were significantly upregulated and 5496 genes were significantly downregulated in the *A. chinensis* hairy roots treated with 5-MI (Figure 3b, Supplementary Table S3).

**Figure 4:**
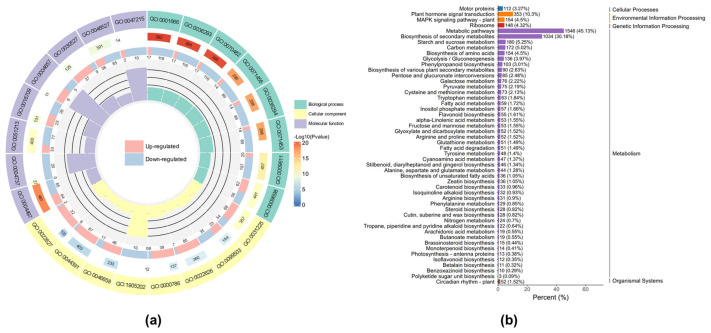
GO and KEGG enrichment analyses of effects of 5-MI treatment on the transcriptome of *A. chinensis* hairy roots. (**a**) GO enrichment analysis of DEGs. The outermost circle represents GO term IDs, with colors indicating the ontology category to which each term belongs. The second circle indicates the total number of genes associated with each term, with colors representing −log_10_ (*p*-value). The third circle displays the number of up- and down-regulated genes, and the fourth circle indicates the Rich factor. The innermost circle provides the legend information. (**b**) KEGG pathway classification of DEGs.

Enrichment analysis was performed by aligning the DEGs to the GO database, and a total of 9074 genes were annotated into 4669 GO terms. As shown in Figure 4a, the DEGs were mainly enriched in the following terms: in the Biological Process category—response to hypoxia, response to decreased oxygen levels, response to oxygen levels, cellular response to hypoxia, and cellular response to decreased oxygen levels; in the Cellular Component category—anchored component of membrane, secretory vesicle, cytosolic ribosome, nucleosome, and methylcrotonoyl-CoA carboxylase complex; and in the Molecular Function category—monooxygenase activity, pyruvate decarboxylase activity, dioxygenase activity, oxidoreductase activity acting on paired donors with incorporation or reduction of molecular oxygen, NAD(P)H as one donor, and incorporation of one atom of oxygen.

The results of the KEGG pathway annotation of the DEGs showed that a total of 3426 genes were enriched in 147 pathways. The top 50 KEGG pathways with the lowest q-value in the enrichment analysis were selected (Figure 4b). The pathways with the largest number of enriched DEGs were mainly related to metabolism, including metabolic pathways, biosynthesis of secondary metabolites, starch and sucrose metabolism, carbon metabolism, biosynthesis of amino acids, phenylpropanoid biosynthesis, galactose metabolism, pyruvate metabolism, cysteine and methionine metabolism, and tryptophan metabolism. In addition, DEGs were also enriched in motor proteins in the Cellular Processes category; plant hormone signal transduction and MAPK signaling pathway—plant in the Environmental Information Processing category; ribosome in the Genetic Information Processing category; and circadian rhythm—plant in the Organismal Systems category.

These results indicate that 5-MI treatment significantly affected the expression of genes related to metabolic regulation and secondary metabolite biosynthesis in *A. chinensis* hairy roots, which provides important clues for further analyzing the molecular mechanism by which 5-MI regulates terpenoid metabolism.

### 2.4. Analysis of Genes Related to Terpenoid Biosynthesis

**Figure 5:**
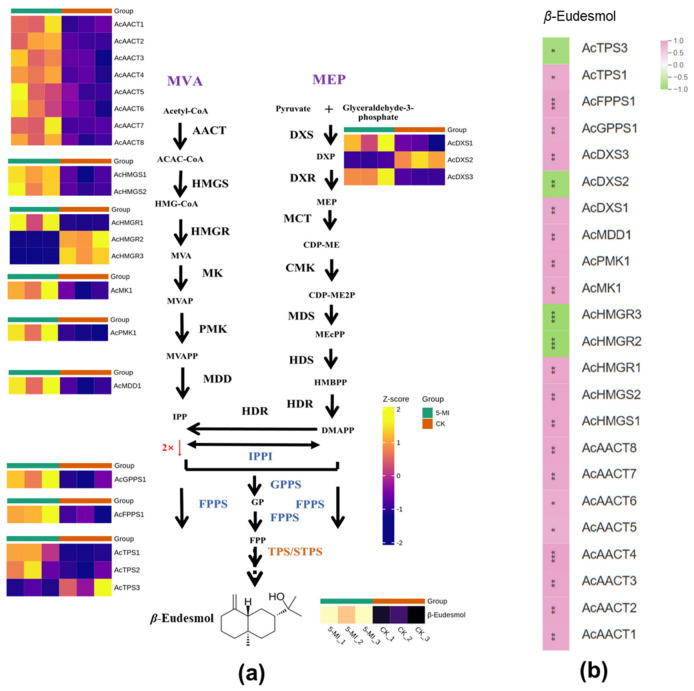
Analysis of genes related to terpenoid biosynthesis in *A. chinensis* hairy roots under 5-MI treatment. (**a**) Schematic diagram of the terpenoid biosynthesis pathway, including the mevalonate (MVA) pathway and the 2-C-methyl-D-erythritol 4-phosphate (MEP) pathway. The expression levels of genes are shown in the heatmap. Dotted lines indicate multiple enzyme-catalyzed processes. The normalized values are presented on the color scale. (**b**) Correlation analysis between the 24 identified terpenoid biosynthesis-related genes and *β*-eudesmol content. Three independent biological replicates were used, and asterisks (*) indicate a significant difference (*p* < 0.05), ** indicate *p* < 0.01, and *** indicate *p* < 0.001 Pink represents a positive correlation, and green represents a negative correlation.

To explore the effect of 5-MI treatment on terpenoid metabolism and the biosynthesis of its downstream derivatives, we focused on the KEGG pathways related to terpenoid biosynthesis, including terpenoid backbone biosynthesis (ko00900), sesquiterpenoid and triterpenoid biosynthesis (ko00909), diterpenoid biosynthesis (ko00904), monoterpenoid biosynthesis (ko00902), and ubiquinone and other terpenoid-quinone biosynthesis (ko00130) (Supplementary Table S4). The backbones of terpenoid compounds are biosynthesized via two isoprenoid biosynthesis pathways, the mevalonate (MVA) pathway and the 2-C-methyl-D-erythritol 4-phosphate (MEP) pathway [25]. A total of 24 genes involved in the terpenoid biosynthesis pathway were identified, and terpenoids were mainly synthesized through the MVA pathway (Figure 5a). In the MVA pathway, eight genes encoding acetyl-CoA C-acetyltransferase (AACT), two genes encoding hydroxymethylglutaryl-CoA synthase (HMGS), three genes encoding hydroxymethylglutaryl-CoA reductase (HMGR), one gene encoding mevalonate kinase (MVK), one gene encoding phosphomevalonate kinase (PMK), and one gene encoding mevalonate diphosphate decarboxylase (MDD) were identified. Meanwhile, only three genes encoding 1-deoxy-D-xylulose-5-phosphate synthase (DXS) were identified in the MEP pathway. Downstream of the MVA and MEP pathways, one gene encoding geranyl diphosphate synthase (GPPS), one gene encoding farnesyl diphosphate synthase (FPPS), and three genes encoding terpene synthase (TPS) were identified [26]. Most of the key genes in the terpenoid biosynthetic pathway were upregulated in *A. chinensis* hairy roots under 5-MI treatment (Figure 5a). Correlation analysis was performed between these 24 genes and the *β*-eudesmol metabolite. The results showed that 23 genes had a strong correlation with *β*-eudesmol, except for the *AcTPS2* gene. Among them, *AcHMGR2*, *AcHMGR3*, *AcDXS2*, and *AcTPS3* were negatively correlated with *β*-eudesmol, while the remaining genes were positively correlated with β-eudesmol (Figure 5b).

**Figure 6:**
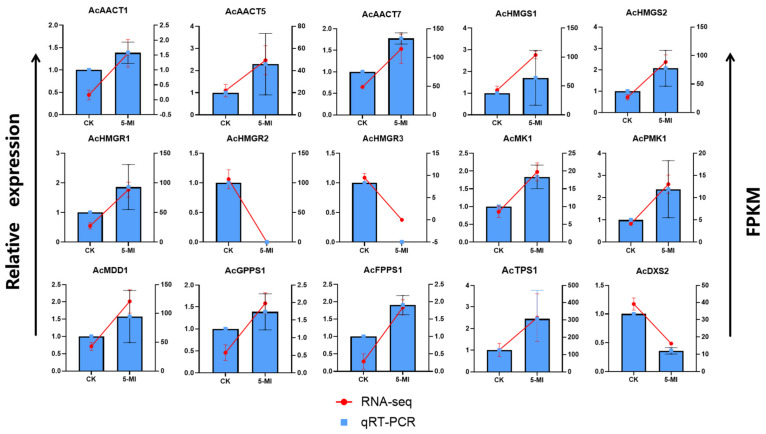
Validation of key gene expression in the terpenoid biosynthesis pathway by qRT-PCR. On the left y-axis, the expression data obtained by qRT-PCR are represented by blue bar graphs, while on the right y-axis, the relative gene expression levels measured by RNA-seq are represented by red lines. *Ac26S* was used as the reference gene for normalization. The qRT-PCR expression level of all genes in the CK group was set to 1.

The RNA-Seq analysis results showed that 5-MI treatment significantly upregulated the expression level of *AcAACT* genes at 7 dpt. Further validation by RT-qPCR revealed that the expression of six *AcAACT* genes (*AcAACT1*), two *AcHMGS* genes (*AcHMGS1-2*), *AcHMGR1*, *AcMK1*, *AcMDD1*, *AcGPPS1*, *AcFPPS1*, two *AcDXS* genes (*AcDXS1* and *AcDXS3*), and *AcTPS1* was significantly enhanced after 5-MI treatment (Figure 6). The changes in the expression patterns of these genes indicate that 5-MI treatment significantly affected the expression of key upstream genes in terpenoid metabolism in *A. chinensis* hairy roots. These results demonstrate that 5-MI treatment induced changes in terpenoid metabolism in *A. chinensis* hairy roots at 7 dpt.

### 2.5. Cloning and Functional Analysis of Terpene Synthase AcTPS1

**Figure 7:**
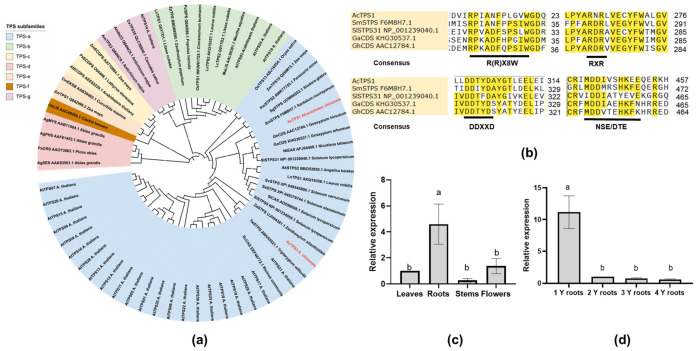
Functional analysis of terpene synthase gene *AcTPS1*. (**a**) Phylogenetic tree of AcTPS1 and TPS proteins from multiple plant species. (**b**) Multiple sequence alignment of AcTPS1 with other TPS proteins, containing the R(R)X8W, RXR, DDXXD, and NSE/DTE motifs of TPS proteins. (**c**) Relative expression levels of *AcTPS1* in roots, stems, flowers, and leaves of two-year-old *A. chinensis* plants. (**d**) Relative expression levels of *AcTPS1* in roots of one-year-old, two-year-old, three-year-old, and four-year-old *A. chinensis* plants. Lowercase letters indicate significant differences among different groups, determined by one-way ANOVA followed by Duncan’s post hoc test (*p* < 0.05). *Ac26S* was used as the reference gene for normalization. The relative expression level of *AcTPS1* in leaves or two-year-old roots was set to 1.

After 5-MI treatment, the expression level of the terpene synthase gene *AcTPS1* in *A. chinensis* hairy roots was significantly upregulated, and it was likely involved in *β*-eudesmol biosynthesis. To further investigate the function of this gene, we cloned and obtained the full-length sequence of *AcTPS1* (Supplementary Figure S3). The full-length ORF of *AcTPS1* is 1644 bp. A phylogenetic tree was constructed based on the sequences of multiple TPS proteins (Figure 7a). The phylogenetic tree divided the TPS proteins into TPS-a to TPS-g subfamilies, and AcTPS1 belongs to the TPS-a subfamily. From the perspective of amino acid sequence evolution, AcTPS1 is most closely related to SmSTPS (F6M8H7.1) from *S. murrayanum*. Subsequently, multiple sequence alignment was performed between AcTPS1 and TPS proteins from *S. lycopersicum* (SlSTPS31), *S. murrayanum* (SmSTPS), *G. arboreum* (GaCDS), and *G. hirsutum* (GhCDS). The alignment results showed that AcTPS1 has the conserved structural characteristics of class a TPS, including the R(R)X8W, RXR, DDXXD, and NSE/DTE motifs (Figure 7b) [27].

The first-strand cDNA from root, stem, leaf, and flower tissues of two-year-old *A. chinensis* plants was used as a template to analyze the expression levels of the *AcTPS1* gene in different tissues. The results showed that, with *AcTPS1* expression in leaves as the reference, the expression level of *AcTPS1* was the highest in the roots, being 4.6-fold higher than in the leaves. There was no significant difference in *AcTPS1* in the stems, leaves, and flowers of two-year-old *A. chinensis* (Figure 7c). In addition, the expression of *AcTPS1* in the roots of *A. chinensis* at different growth years (one, two, three, and four years old) was examined. The results showed that, with *AcTPS1* expression in two-year-old roots as the reference, *AcTPS1* was highly expressed in one-year-old roots of *A. chinensis*, being 19.5-fold higher than in two-year-old roots. There was no significant difference in the expression level of *AcTPS1* in two-, three- and four-year-old roots (Figure 7d).

## 3. Discussion

The conservation of medicinal plant germplasm resources mainly includes seed propagation, artificial cultivation, plant tissue culture, and hairy root culture. Medicinal plants produce a variety of phytochemicals such as cyclic peptides, quinones, alkaloids, flavonoids, and terpenes, which are widely used in the pharmaceutical and fragrance industries [28,29]. The main commercial source of these metabolites is field-grown plants, which are often affected by seasonal changes and regional differences, thus restricting the content of secondary metabolites in medicinal plants. Hairy root culture is an alternative method for the industrial production of high-value secondary metabolites, which can achieve large-scale propagation in a relatively short period of time [30]. While hairy roots are a powerful platform, they may lack certain organ-specific biosynthesis pathways or precursor pools present in intact plants, which can limit the production of some metabolites. As an important medicinal plant, our team previously established hairy roots of *A. chinensis*. To increase the content of β-eudesmol in *A. chinensis* hairy roots, this study explored the application of chemical elicitors. The success of the 5-MI elicitation strategy demonstrates that it can serve as a powerful biotechnological approach for overcoming this limitation by artificially activating the existing pathways in the hairy root system.

Although 5-MI has been rarely studied as an elicitor, its chemical homolog MT is a well-recognized chemical elicitor known to promote the accumulation of secondary metabolites in a variety of plants [6]. Treatment of cabbage with 100 μmol·L^−1^ MT upregulated anthocyanin biosynthesis genes and stimulated anthocyanin accumulation [31]. MT-mediated nitric oxide production enhanced isoflavone accumulation by increasing the activities of phenylalanine ammonia-lyase and cinnamate 4-hydroxylase and upregulating key biosynthetic genes [32]. However, in our previous experiments, treatment with different concentrations of MT failed to significantly induce the accumulation of *β*-eudesmol in *A. chinensis* hairy roots [16]. In contrast, treatment with 0.5 mmol·L^−1^ 5-MI significantly increased the content of *β*-eudesmol to 16.91 mg·g^−1^, indicating that 5-MI can be used as an effective elicitor to significantly increase the content of the key active component *β*-eudesmol in *A. chinensis* hairy roots. The mechanistic basis for this differential efficacy remains unknown. This difference may be attributed to the biological differences between hairy roots and intact plants. Reactive oxygen and nitrogen species (ROS/RNS) are established second messengers in elicitor-triggered secondary metabolism [33]. While it is possible that 5-MI perception similarly activates ROS/RNS signaling to promote sesquiterpenoid accumulation, this hypothesis requires direct experimental validation in future studies.

To further explore the underlying mechanism, we performed integrated transcriptomic and metabolomic analyses on *A. chinensis* hairy roots treated with 0.5 mmol·L^−1^ 5-MI for 7 days. Through a widely targeted metabolomic analysis, a total of ten upregulated terpenoid metabolites were identified in the DEMs after 5-MI treatment, including sesquiterpenoids such as *β*-eudesmol. In addition, the related DEMs were enriched in the diterpenoid biosynthesis metabolic pathway related to terpenoid biosynthesis. Therefore, treatment with 0.5 mmol·L^−1^ 5-MI can significantly affect the metabolism of substances in *A. chinensis* hairy roots and induce extensive changes in metabolites. Although 5-MI has been rarely studied as an elicitor, treatment with its structural analog MT has been reported to significantly affect the terpenoid metabolic pathway, especially the biosynthesis and accumulation of monoterpenes and sesquiterpenes [34].

Transcriptome sequencing helps to elucidate the changes in gene expression levels and their correlation with metabolite changes between different treatment groups. Through transcriptome sequencing analysis, this study identified DEGs induced by 5-MI treatment, which provided a theoretical basis for the changes in metabolites in *A. chinensis* hairy roots. The present transcriptomic analysis, while rigorous and informative, was performed on bulk hairy root tissue, which inherently masks the cell-type heterogeneity that may be crucial for terpenoid biosynthesis. Hairy roots are complex multicellular structures comprising distinct cell types (e.g., epidermal, cortical, endodermal, pericycle, and vascular cells), each of which may contribute differently to the production of sesquiterpenoids [35]. Recent advances in single-cell and spatial transcriptomics have revolutionized our ability to resolve cell-type-specific transcriptional programs in plant roots, providing unprecedented insights into the spatial organization of secondary metabolic pathways [36]. In the context of hairy roots, which are characterized by asynchronous growth and heterogeneous cell populations, bulk RNA-seq may obscure the contributions of rare cell types that are the primary sites of sesquiterpenoid biosynthesis. The biosynthesis of terpenoids is carried out through the MVA and MEP pathways, which function in the cytoplasm and plastids, respectively [25]. Previous studies have found that the activation degree of these two pathways differs in plants exposed to abiotic or biotic stresses. Zhou et al. [37] and Wang et al. [38] found that the activation degree of the MVA pathway was higher than that of the MEP pathway in *Atractylodes macrocephala* and *A. lancea* under the treatment of biotic elicitors, respectively. Consistent with these findings, the present study showed that the activation degree of the MVA pathway was higher than that of the MEP pathway after induction by 5-MI treatment. In the MVA pathway, eight genes encoding AACT, two genes encoding HMGS, three genes encoding HMGR, one gene encoding MVK, one gene encoding PMK, and one gene encoding MDD were identified [39]. In contrast, only three genes encoding DXS were identified in the MEP pathway, with *AcDXS1* (log_2_FC 1.01–1.07) showing increased expression. In this study, the increased expression levels of *AcAACTs* (log_2_FC 1.01–3.22), *AcHMGSs* (log_2_FC 1.13–1.57), *AcHMGR1* (log_2_FC 1.65), *AcMVK1* (log_2_FC 1.06), *AcPMK1* (log_2_FC 1.52), and *AcMDD1* (log_2_FC 1.36) indicated the accumulation of their corresponding products, and these precursors may be preferentially directed to biosynthesis via the MVA pathway. Previous studies have identified HMGR as the rate-limiting enzyme in the MVA pathway [40]. In this study, one *HMGR* gene (*AcHMGR1*) was found to be significantly upregulated after 5-MI treatment. The determination of metabolite content showed that the contents of sesquiterpenoids, including dictyosporin C, α-curcumene, and *β*-eudesmol, were significantly increased after 5-MI treatment. The upregulated expression of key genes involved in terpenoid biosynthesis, including *AcGPPS1*, *AcFPPS1*, and *AcTPS1*, further supported this result [41].

These results strongly support the notion that 5-MI treatment activates sesquiterpenoid biosynthesis. In this study, we also found that the expression of the terpene synthase gene *AcTPS1* was significantly upregulated after 5-MI treatment, which was positively correlated with the content of β-eudesmol. The TPS family is closely related to the biosynthesis of terpenoids, which includes seven subfamilies: TPS-a to TPS-g [42]. In this study, the *AcTPS1* gene, which encodes a sesquiterpene synthase and belongs to the TPS-a subfamily, was cloned. Previous studies have shown that the TPS-a subfamily contains the vast majority of sesquiterpene and diterpene synthases [43]. Plant terpene synthases (TPSs) catalyze the first committed step in terpenoid diversification and typically employ a class I mechanism that relies on conserved metal-binding motifs [42]. TPS-a subfamilies utilize a tri-nuclear metal cluster coordinated by two conserved DDXXD and NSE/DTE motifs. The DDXXD motif is essential for binding three Mg^2+^ or Mn^2+^ ions that coordinate the pyrophosphate group of the allylic diphosphate substrate, while the NSE/DTE motif contributes to additional metal coordination and stabilization of the carbocation intermediate during the cyclization cascade [43]. The AcTPS1 protein contains both the DDXXD and NSE/DTE motifs, as revealed by multiple sequence alignment, strongly suggesting that AcTPS1 employs a classical class I metal-dependent catalytic mechanism to convert its presumptive substrate, farnesyl diphosphate (FPP), into sesquiterpenoid products. Terpene synthases have been studied in a variety of plants, and the volatile oil of *Atractylodes* species contains a variety of sesquiterpenoids [44]. The biosynthesis of *β*-eudesmol in turmeric (*Curcuma longa*) has been attributed to *ClTPS15*, which specifically converts FPP to *β*-eudesmol [45]. Similarly, in *A. lancea*, two TPS-a subfamily members, *AlTPS13* and *AlTPS20*, were shown to use FPP as a substrate to produce a spectrum of sesquiterpenoids, although the specific product profiles remain to be fully characterized [44]. Given the high sequence similarity and shared conserved motifs among *AcTPS1*, *ClTPS15*, and *AlTPS13/20*, it is plausible that *AcTPS1* catalyzes FPP cyclization to yield *β*-eudesmol or its immediate precursors. However, definitive confirmation of the enzymatic function of *AcTPS1* would require heterologous expression (e.g., in *E. coli* or yeast) followed by in vitro enzyme assays with FPP and GC-MS product profiling. Terpene synthase genes exhibit organ-specific expression and are regulated by plant development [46]. The higher expression of *AcTPS1* in roots than in leaves contributes to the understanding of the regulation of organ- and tissue-specific metabolites in *A. chinensis*, while the upstream regulatory mechanism remains unclear. Our subsequent research will focus on analyzing the promoter cis-acting elements of *AcTPS1* and screening the upstream transcription factors so as to resolve the molecular regulatory mechanism of its spatiotemporal specific expression.

## 4. Materials and Methods

### 4.1. Plant Material

Hairy roots of *Atractylodes chinensis* (DC.) Koidz were maintained in our laboratory and cultured in 1/2 MS medium (90 mL in 150 mL flasks) supplemented with 0.5 mg·L^−1^ indole acetic acid (IAA), 0.1 mg·L^−1^ kinetin (KT), and 5% sucrose, with shaking at 120 rpm and 25 °C [16]. Hairy roots cultured for 52 days were transferred to fresh medium containing 0.5 mmol·L^−1^ 5-MI (Solarbio, Beijing, China) on day 0. And the hairy roots were harvested 7 days post-treatment (dpt) without any intermediate medium change. Samples were snap-frozen in liquid nitrogen and stored at −80 °C for subsequent metabolomic and transcriptomic analyses, with three independent biological replicates set for each group.

### 4.2. Determination of β-Eudesmol Content

The treated hairy roots were thoroughly rinsed, dried at 37 °C, and ground into fine powder. For *β*-eudesmol determination, 0.2 g of the powder was extracted with 4 mL of 70% methanol for 12 h. The extracts were centrifuged at 10,000× *g* for 8 min, and the supernatant was collected. After filtration through a 0.22 μm PTFE membrane, samples were analyzed using an ultra-performance liquid chromatography (UPLC) system. The gradient elution conditions were maintained at 32% solvent A (water) and 68% solvent B (acetonitrile) at a flow rate of 0.45 mL·min^−1^ and a detection wavelength of 203 nm from the initial time to 3.00 min without any adjustment [47]. And *β*-eudesmol was quantified based on the standard curve.

### 4.3. Metabolomic Analysis

After freeze-drying and grinding, samples were extracted with 70% methanol. Following centrifugation at 12,000 rpm for 3 min, the supernatant of the samples was filtered through a 0.22 μm PTFE for UPLC–tandem mass spectrometry (UPLC-MS/MS) analysis, performed by Metware Biotechnology Co., Ltd. (Wuhan, China). Metabolite identification was achieved by matching MS/MS spectra against the in-house MWDB database. For quantification, MRM mode was used: the first quadrupole selected the precursor ion, the collision cell generated fragment ions, and the third quadrupole selected a characteristic fragment ion. The peak areas of all metabolites were integrated and normalized using the internal standard. The OPLS-DA model was tested with 200 permutations. The Q^2^ and R^2^ values were reported, confirming model validity. Differentially expressed metabolites (DEMs) between the 5-MI treatment group and the control (CK) group were screened with the screening criteria of variable importance in projection (VIP) ≥ 1, fold change (FC) ≥ 1.5 or FC ≤ 0.75, and *p* value < 0.05. KEGG enrichment analysis was performed using the Metware Cloud, a free online platform for data analysis (https://cloud.metware.cn, URL accessed on 25 December 2025).

### 4.4. Transcriptomic Analysis

The total RNA of all samples was extracted. RNA quality was assessed using an Agilent 2100 Bioanalyzer (RIN ≥ 7.0). Strand-specific libraries were constructed using an NEBNext Ultra RNA Library Prep Kit (NEB, Whatman, USA). Strand-specific sequencing was performed on the NovaSeq 6000 platform by Metware Biotechnology Co., Ltd. (Wuhan, China) with 150 bp paired-end reads, generating approximately 12 Gb of raw data per sample. Adapter trim ming and duplicate removal were performed on the raw sequences obtained from sequencing. After removing adapter sequences and low-quality sequences from the raw data, high-quality clean reads were obtained. De novo assembly of the clean reads was performed using Trinity (v2.8.5), and further sequence assembly and redundancy removal were conducted using the sequence clustering software Corset (https://bioinformaticshome.com/db/tool/corset/, accessed on 18 December 2025) to obtain polished consensus sequences. Gene expression levels were quantified as FPKM (Fragments Per Kilobase of transcript per Million mapped reads) using RSEM (v1.3.1).

Differential gene expression analysis was performed following the standard procedures of Metware Biotechnology Co., Ltd. (Wuhan, China). Differentially expressed genes (DEGs) were identified with the screening criteria of |log_2_Fold Change| ≥ 1 and false discovery rate (FDR) < 0.05. Functional annotation of the genes was performed against multiple databases, including NR, Swissprot, GO, Clusters of Orthologous Groups (COG), euKaryotic Orthologous Groups (KOG), KEGG, and Pfam. BLAST (v2.14.0) software was used to align the Unigene sequences with the NR, Swiss-Prot, GO, COG, KOG, and KEGG databases. After predicting the amino acid sequences of the Unigenes, HMMER (v3.3.2) software was used to align the sequences with the Pfam database to obtain the annotation information of the Unigenes. The transcripts assembled and de-redundant by Trinity were used as the reference sequence, and the clean reads of each sample were mapped to the reference sequence. Based on the mapping results, the read count number of each sample mapped to each transcript and gene was obtained in combination with RSEM and converted to a Fragments Per Kilobase of transcript per Million mapped reads (FPKM) value. TransDecoder (V5.0.0) software was used to predict the coding sequence (CDS).

Differential expression analysis was performed based on the quantitative results of gene expression, followed by functional enrichment analysis of the DEGs. DESeq2 was used with Benjamini–Hochberg FDR correction (FDR < 0.05) for DEG identification. After the screening of the DEGs, functional annotation was performed, mainly including GO and KEGG analyses. ClusterProfiler (https://clusterprofiler.com/, accessed on 28 December 2025) was used for the GO functional enrichment analysis and KEGG pathway enrichment analysis of the DEGs. A *p* value < 0.05 was set as the threshold for significant enrichment in both the GO and KEGG analyses, which were performed by using the Metware Cloud, a free online platform for data analysis (https://cloud.metware.cn). We focused on the KEGG pathways related to terpenoid biosynthesis, including terpenoid backbone biosynthesis (ko00900), sesquiterpenoid and triterpenoid biosynthesis (ko00909), diterpenoid biosynthesis (ko00904), monoterpenoid biosynthesis (ko00902), and ubiquinone and other terpenoid-quinone biosynthesis (ko00130), from which the DEGs involved in terpenoid biosynthesis were selected. The correlation analysis between the selected DEGs in terpenoid biosynthesis and *β*-eudesmol was performed with the screening criteria of |R| ≥ 0.8 and *p* < 0.05.

### 4.5. Quantitative Real-Time PCR (qRT-PCR) Analysis and Statistical Analysis

Key genes involved in terpenoid biosynthesis were selected based on KEGG pathways for qRT-PCR validation, and the *Ac26S* gene was used as the internal reference gene. Total RNA was extracted using a MiniBEST Universal RNA Extraction Kit (TaKaRa, Beijing, China). First-strand cDNA was synthesized using a PrimeScript™ RT Reagent Kit with gDNA Eraser (TaKaRa, Beijing, China). The qRT-PCR reaction system and procedures were performed according to the instructions of the TB Green Premix Ex Taq II kit (TaKaRa, Beijing, China). The 2^−ΔΔCt^ method was used to calculate the relative expression levels of genes. Three independent biological replicates were set for each sample. SPSS 23.0 software was used to determine the statistical significance (*p* < 0.05).

### 4.6. Gene Cloning and Functional Analysis

Full-length primers for *AcTPS1* were designed according to the predicted sequence from the transcriptomic data (Supplementary Table S5). PCR amplification was performed using the first-strand cDNA of *A. chinensis* hairy roots as the template to obtain the *AcTPS1* gene sequence. The ORF sequence of *AcTPS1* was analyzed using NCBI ORF Finder (http://www.ncbi.nlm.nih.gov/gorf/gorf.html, accessed on 7 March 2026). The protein sequence of AcTPS1 was analyzed using Protparam (https://www.expasy.org/, accessed on 7 March 2026) [48]. A phylogenetic tree was constructed using the Maximum likelihood tree in MEGA 12.0 software with the protein sequences from the following species: *Zea mays* ZmTPS10 (Q2NM15.1), ZmECDPS (AAT70084.1), *Oryza sativa* OsTPS13 (ABJ16554.1), *Arabidopsis thaliana* AtTPS01-30, AtECDPS (AEE82229.1), *Solanum lycopersicum* SlSTPS9 (NP_001234055.1), SlSTPS31 (NP_001239040.1), *Cinnamomum burmanni* CbTPS1 (WKW91725.1); *Cannabis sativa* CsTPS35 (QLC36840.1), *Litsea cubeba* LcTPS1 (G0Y7D1.1), *Laurus nobilis* LnTPS1 (AKQ19358.1), LnTPS2 (AKQ19357.1), *Zanthoxylum piperitum* ZpTPS (BBD88590.1), *Sindora glabra* SgSTPS (UOW66203.1), *Santalum murrayanum* SmSTPS (F6M8H7.1), *Solanum verrucosum* SvSTPS (XP_049345898.1), *Solanum stenotomum* SsSTPS (XP_049379744.1), *Zanthoxylum ailanthoides* ZaSTPS (UJH94381.1), *Persicaria minor* PmSTPS2 (AWK77755.1), *Angelica keiskei* AkSTPS2 (BBO53955.1), *Tripterygium wilfordii* TwSTPS (AWV55521.1), *Abies grandis* AgMYS (AAB71084.1), AgPHS (AAF61453.1), AgSES (AAK83561.1), *Picea abies* PaCRS (AAO73863.1), *Clarkia breweri* CbLIS (AAC49395.1), *Populus tremula* PcISPS (Q9AR86.1), *Mentha aquatica* MaLIS (AAL99381.1), *Gossypium arboreum* GaCDS (KHG30537.1), *Gossypium hirsutum* GhCDS (AAC12784.1), *Ricinus communis* RcCAS (EEF48772.1), *Nicotiana tabacum* NtEAS (AFJ04408.1), *Antirrhinum majus* Am0e23 (Q84NC8.1), *Cucurbita maxima* CmEKSB (AAB39482.1), and *Lathyrus oleraceus* PsECDPS (O04408.1) [49]. Multiple sequence alignment was performed using clustalo software.

Root, stem, leaf, and flower tissues of two-year-old *A. chinensis* plants were collected to determine the tissue distribution of the *AcTPS1* gene. Root tissues of one-year-old, two-year-old, three-year-old, and four-year-old *A. chinensis* were collected to detect the expression levels of *AcTPS1* at different growth years of *A. chinensis* roots. Three independent biological replicates were used.

## 5. Conclusions

Treatment of *A. chinensis* hairy roots with 5-MI, a structural analog of MT, significantly increased the content of the secondary metabolite *β*-eudesmol. Treatment with 0.5 mmol·L^−1^ 5-MI significantly affected the metabolism of substances in *A. chinensis* hairy roots and induced extensive changes in metabolites. Meanwhile, 5-MI treatment significantly affected the expression of genes related to metabolic regulation and secondary metabolite biosynthesis in *A. chinensis* hairy roots, particularly upregulating the expression of genes involved in terpenoid biosynthesis. Among them, a putative sesquiterpene synthase gene, *AcTPS1*, was significantly upregulated after 5-MI treatment and was a strong candidate gene correlated with *β*-eudesmol. *AcTPS1* belongs to the TPS-a subfamily and exhibits tissue-specific expression with significantly upregulated expression in roots. This study enriches our understanding of the role of 5-MI in the production of plant secondary metabolites.

## Figures and Tables

**Table 1:** Content of *β*-eudesmol in *A. chinensis* hairy roots in control and 5-MI treatment groups.

Content of *β*-Eudesmol (mg/g)		
	CK	5-MI
7 dpt	7.30 ± 0.94 B	16.91 ± 0.58 A ^1^

^1^ The capital letters indicate significant differences among different lines, determined via ANOVA followed by Duncan’s post-test (*p* < 0.01).

**Table 2:** Upregulated terpenoid metabolites after treatment with 5-MI in *A. chinensis* hairy roots compared with the control group.

No.	Formula	Metabolites	CAS	VIP	*p*_Value
1	C_15_H_24_O_2_	Dictyosporin C	not available	1.29	0.05
2	C_21_H_34_O_7_	3,11(13)-Eudesmadiene-1,12-diol 12-O-β-D-Glucopyranoside	135448-10-1	1.39	0.02
3	C_24_H_36_O_10_	7-Hydroxy-costol-malonyl glucoside	not available	1.46	0.02
4	C_21_H_32_O_9_	6-hydroxy-4a-methyl-8-methylidene-8a-[3,4,5-trihydroxy-6-(hydroxymethyl)oxan-2-yl]oxy-2,3,4,5,6,7-hexahydro-1H-naphthalen-2-yl]prop-2-enoic acid	not available	1.42	0.00
5	C_26_H_46_O_12_	3,7,11-trimethyldodeca-3,7-diene-1,10,11-triol 10-O-(beta-D-Xylopyranosyl)-beta-D-glucopyranoside	not available	1.58	0.01
6	C_22_H_34_O_10_	9-Hydroxy-4-Megastigmen-3-one O-(6-O-Malonyl-β-D-Glucopyranoside)	1007227-01-1	1.37	0.01
7	C_10_H_16_O_2_	7-(hydroxymethyl)-2,3,3a,4,5,7a-hexahydro-1H-inden-4-ol	not available	1.58	0.001
8	C_15_H_22_	alpha-Curcumene	4176-17-4	1.58	0.01
9	C_30_H_46_O	Lupa-1,20(29)-dien-3-one (Glochidone)	not available	1.34	0.03
10	C_15_H_26_O	β-Eudesmol	473-15-4	1.25	0.01

## Data Availability

The original contributions presented in this study are included in the article; further inquiries can be directed to the corresponding author.

## Supplementary Materials

The following supporting information can be downloaded at: https://www.mdpi.com/article/10.3390/plants15132027/s1, Table S1: Upregulated terpenoid metabolites in *A. chinensis* hairy roots after treatment with 5-MI. Table S2: Summary data of RNA-seq. Table S3: Differential expression genes and effects of 5-MI treatment on the metabolome of *A. chinensis* hairy roots. Table S4: Differential gene expression in terpenoid biosynthesis pathways. Table S5: Primer sequences used in this study. Figure S1: OPLS-DA verification diagram of metabolomics. Figure S2: UPLC chromatograms of upregulated terpenoid metabolites in *A. chinensis* hairy roots after treatment with 5-MI compared with the control group. Figure S3: The molecular cloning of *AcTPS1*.
