# Supplementary material for: Integrated Transcriptomic and Metabolomic Analyses Reveal the Mechanism by Which 5-Methoxyindole Enhances Sesquiterpenoids Production in Atractylodes chinensis Hairy Roots
Source: Plants (Basel). 2026 Jun 30;15(13):2027. doi: 10.3390/plants15132027 (PMC13364421; doi:10.3390/plants15132027)
Supplement: Supplementary file 1 [file plants-15-02027-s001.zip › Supplementary Figure.pdf]

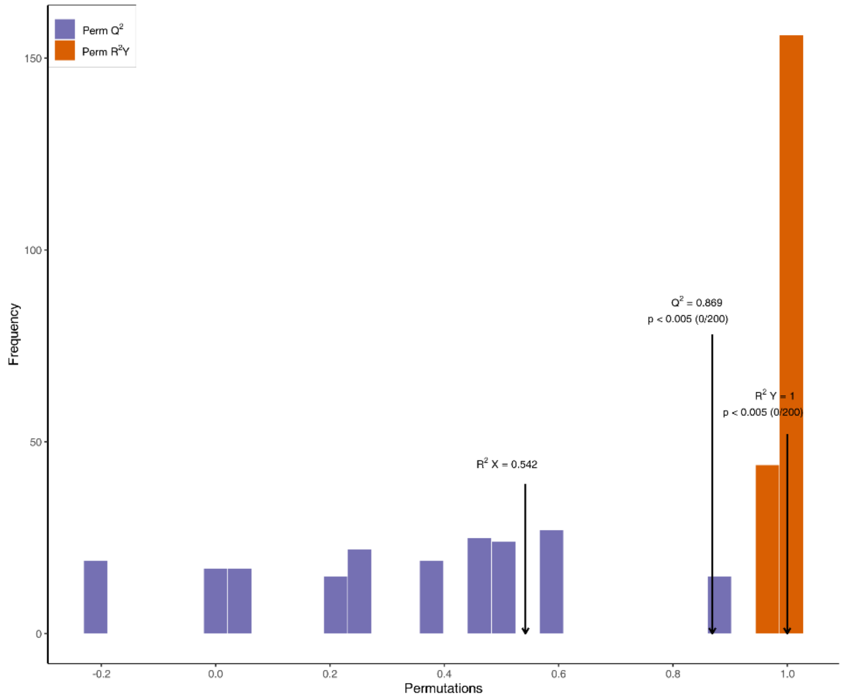

Figure S1 OPLS-DA Verification Diagram of Metabolomics.

The horizontal axis represents the R^2Y and Q^2 values of the model, and the vertical axis represents the model in 200 random permutation and combination experiments. The frequency of appearance of classification effects. The orange in the figure represents the random grouping model R^2Y, and the purple represents randomness. Grouping model Q^2, the black arrows represent the R^2X, R^2Y, and Q^2 values of the original model.

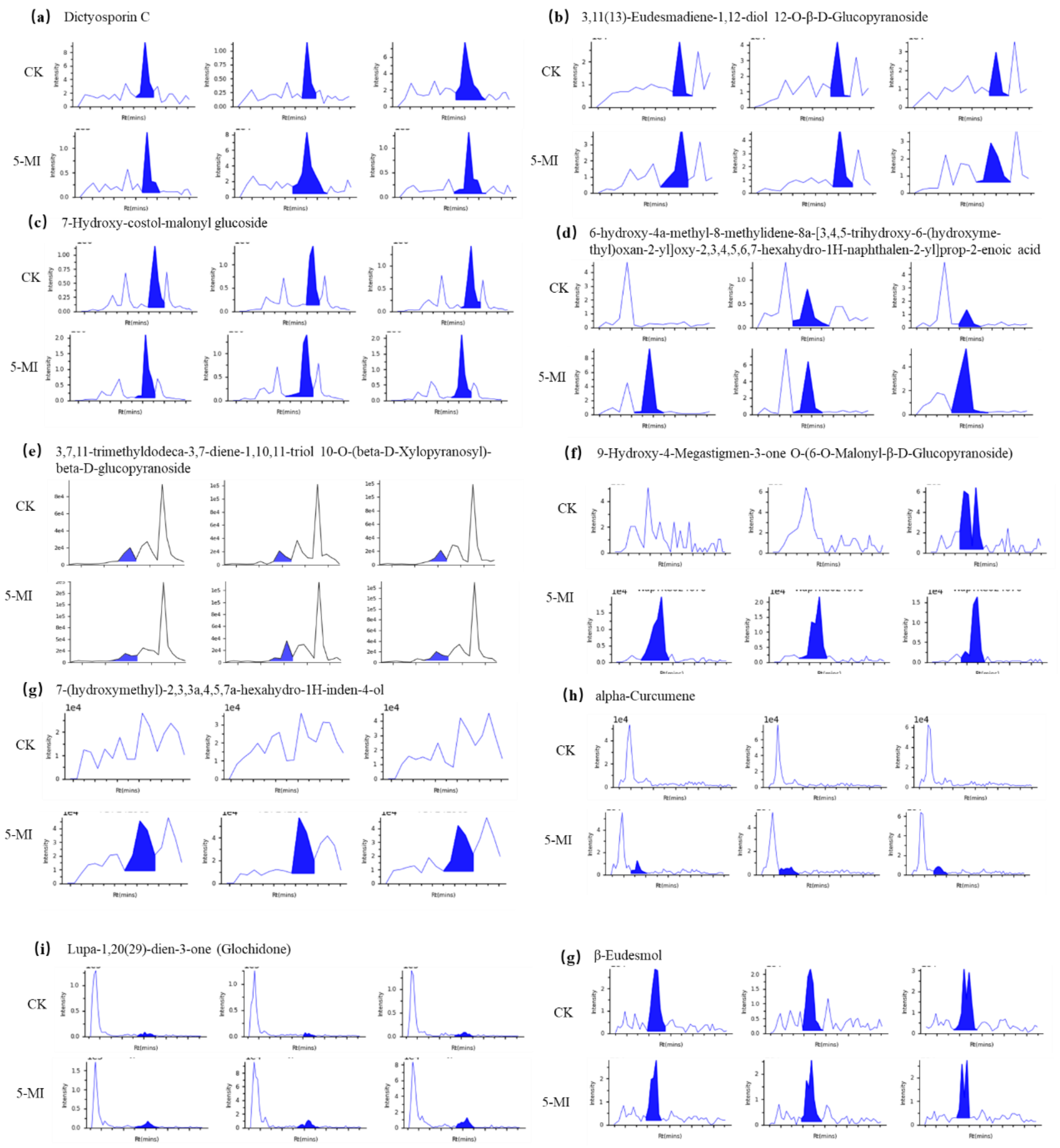

Figure S2 The UPLC chromatograms of upregulated terpenoid metabolites in treatment with 5-MI A. chinensis hairy roots compared with the control group.

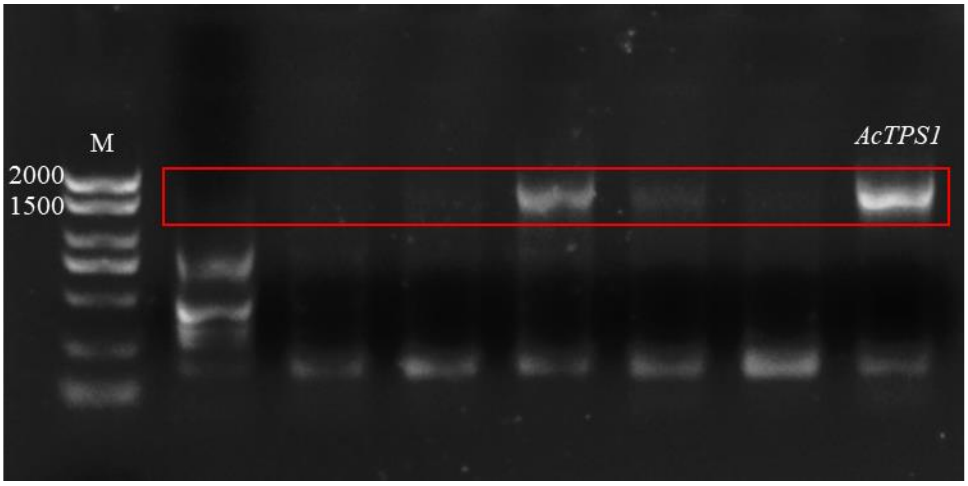

Figure S3 The molecular cloning of *AcTPS1*.
